# Comparison of arterial input function measured from dual-bolus and dual-sequence dynamic contrast-enhanced cardiac magnetic resonance imaging

**DOI:** 10.1186/1532-429X-13-S1-O8

**Published:** 2011-02-02

**Authors:** Li-Yueh Hsu, Peter Kellman, Peter Gatehouse, Sven Zuehlsdorff, Christopher B Glielmi, Daniel W Groves, Anthony H Aletras, Patricia W Bandettini, Andrew E Arai

**Affiliations:** 1 National Institutes of Health, Bethesda, MD, USA; 2 Royal Brompton Hospital, London, UK; 3 Siemens Medical Solutions, Chicago, IL, USA

## Introduction

Estimates of myocardial blood flow (MBF) from first-pass contrast-enhanced cardiac magnetic resonance (CMR) imaging require accurate measurement of the arterial input function (AIF) from the left-ventricular (LV) blood pool. Both dual-bolus and dual-sequence CMR techniques have been proposed to maintain the linearity of the LV signal during the contrast passage.

## Purpose

This study is to directly compare the AIF measured from dual-bolus and dual-sequence CMR techniques. Quantitative MBF estimates were compared to microsphere reference using both AIF measurements.

## Methods

Both the dual-sequence CMR perfusion technique [1] and the dual-bolus protocol [2] were performed in five canines and 35 clinical subjects using a 1.5 Tesla scanner. Gd-DTPA at 0.005 mmol/kg and 0.05 mmol/kg were administered during separate breath holds. Typical imaging parameters: 1 RR, 90° saturation pulse, 50°read out angle, saturation recovery 90 ms, TR 2.4 ms, TE 1.2 ms, matrix size 128x80. A low TE, low resolution FLASH image (TE 0.6 ms, matrix size 64x48) was acquired during each RR interval. AIF measured from the low-dose high-resolution (i.e. dual-bolus) image series and high-dose low-resolution (i.e. dual-sequence) image series were compared. Reference MBF measurements were obtained from the canine experiments with intracoronary adenosine infusion and microspheres injection. CMR derived MBF estimates were calculated separately using both AIF by a model-constrained deconvolution. Clinical CMR perfusion studies (35 rest and 35 stress) were also analyzed to compare the AIF from both techniques.

## Results

**Figure 1:**
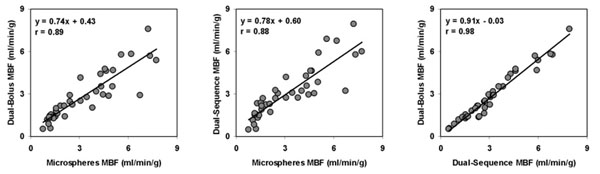


**Figure 2:**
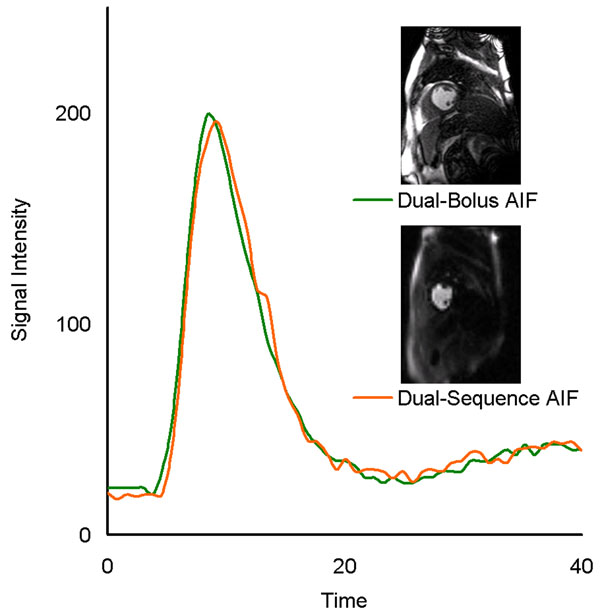


MBF estimates from dual-bolus and dual-sequence CMR techniques matched well with the microspheres measurements (r=0.89 and 0.88, figure-1). There was an excellent agreement between dual-bolus and dual-sequence MBF estimates (r=0.98, figure-1). For clinical perfusion, linear regression analysis shows there was an excellent match between dual-bolus and dual-sequence AIF measurements (figure-2). Pearson correlation coefficient averaged 0.99±0.01 in rest and stress studies.

## Conclusions

MBF estimates using both dual-bolus and dual-sequence CMR techniques matched well with microsphere reference in animal data. AIF measured from both techniques also correlated closely in all clinical perfusion studies. Either a dual-bolus or dual-sequence technique can accurately depict the AIF in first-pass CMR perfusion imaging for MBF quantification. The dual-sequence technique eliminates the need for complex contrast administration, as well as the need for two separate acquisitions. However, the dual-bolus technique does not require a special perfusion sequence and has a shorter acquisition time per RR interval.

